# A descriptive analysis of cross-sectional imaging findings in patients after non-traumatic sudden cardiac arrest

**DOI:** 10.1016/j.resplu.2021.100077

**Published:** 2021-01-28

**Authors:** Charles W Hwang, Muhammad Abdul Baker Chowdhury, Dru Z Curtis, Jon D Wiese, Apara Agarwal, Brandon P Climenhage, Torben K Becker

**Affiliations:** Department of Emergency Medicine UF Health, 1329 SW 16th Street PO Box 100186 Gainesville, FL 32610-0186, USA

**Keywords:** Cross-Sectional imaging, Sudden cardiac arrest, Resuscitation, Post-Cardiac arrest management, ACLS, advanced cardiac life support, ACS, acute coronary syndrome, CT, computed tomography, eCPR, extracorporeal cardiopulmonary resuscitation, ED, emergency department, MR, Imagnetic resonance imaging, OHCA, out-of-hospital cardiac arrest, PEA, pulseless electrical activity, ROS, Creturn of spontaneous circulation, SCA, sudden cardiac arrest, VF, ventricular fibrillation, VT, ventricular tachycardia, WBCT, whole body computed tomography

## Abstract

**Introduction:**

Cross-sectional imaging is frequently obtained after sudden cardiac arrest (SCA) to determine the aetiology. Although imaging studies may reveal acute and/or chronic findings that may impact downstream medical management, lack of standardized guidelines results in significant practice variability. We aimed to perform a descriptive analysis and to report on radiographic findings after SCA.

**Methods:**

This was a retrospective observational descriptive study that included all adult SCA patients who presented to our emergency department (ED) over a 6-year period, achieved sustained return of spontaneous circulation, and subsequently received cross-sectional imaging while in the ED. Each imaging study was reviewed and graded based on a predefined scale, and significant radiographic findings were tabulated.

**Results:**

1573 patients were identified, and 452 patients remained after applying predefined exclusion criteria. A total of 298, 184, and 113 computed tomography (CT) studies were performed of the head, chest, and abdomen, respectively. For head, chest, and abdominal imaging, 13 (4.4%), 23 (12.5%), and 6 (5.3%) studies had radiographic findings that likely contributed to SCA, respectively. Altogether, 42 (7.1%) radiographic studies had findings that likely contributed to SCA. Eighty (13.4%) studies (head [n = 38, 12.8%], chest [n = 26, 14.1%], abdomen [n = 16, 14.2%]) resulted in a change of clinical care (e.g. specialty consultation or procedures).

**Conclusion:**

Given the clinical uncertainty and relative instability during the post-SCA phase, cross-sectional imaging frequently reveals important acute and chronic diagnostic findings.

## Introduction

Out-of-hospital cardiac arrest (OHCA) remains a leading cause of morbidity and mortality in industrialized countries, affecting approximately 350,000 patients per year in the United States,[1], [2], [3], [4]^,^^5^ with an annual incidence of 98.1 per 100,000 person-years, and accounting for 15-20% of all deaths.^6^ Despite advances in research and the implementation of changes to the “chain of survival”, including public education and early recognition, implementation of resuscitation protocols, and advancements in post-cardiac arrest care, the rate of survival remains low at 10.4%.[1], [7], [8], [9]^,^[10], [11]

In the resuscitation and post-arrest phase, determining the aetiology of cardiac arrest is of paramount importance in subsequent management.[1], [2], [12] Acute coronary syndrome (ACS) is a main cause of OHCA, and current guidelines recommend urgent coronary angiography for patients suspected of having OHCA due to cardiac origin.[1], [13], [14], [15]^,^[16], [17], [18], [19]^,^^20^

Therefore, non-cardiac causes, which account for 22-34% of OHCA cases, and their relationship to cardiac arrest have not been as rigorously studied. Non-cardiac causes include conditions such as seizure, subarachnoid haemorrhage, any condition that causes severe metabolic acidosis, haemorrhage, overdose, thromboembolic disease, and trauma, and can present as both shockable (ventricular fibrillation [VF] or ventricular tachycardia [VT]) and non-shockable (asystole or pulseless electrical activity [PEA]) cardiac arrest rhythms.[1], [12], [21], [22]^,^[23], [24], [25] The expedient and accurate diagnosis of non-cardiac causes is important, as patients with these conditions require rapid interventions in the emergency department (ED); identifying these conditions may require additional diagnostic laboratory or radiographic evaluation.

The American Heart Association (AHA), however, has issued no guidelines informing the use of non-invasive diagnostic cross-sectional imaging (e.g. computed tomography [CT] or magnetic resonance imaging [MRI]) to identify other causes of OHCA.[18], [26], [27] While urgent non-invasive imaging is frequently obtained in post-cardiac arrest patients, a lack of standardized guidelines has resulted in significant variability in practice and outcomes at individual centres around the world.^28^ Consequently, the International Liaison Committee on Resuscitation (ILCOR) has stressed the importance of aetiological research focusing on reversible causes.[1], [19]

The purpose of this study was to perform a retrospective observational descriptive analysis and report on ED diagnostic non-invasive cross-sectional imaging findings after OHCA.

## Methods

### Study setting

The UF Health Emergency Department is a large academic, university-based 66-bed ED, which is a tertiary referral centre affiliated with the University of Florida, with a full complement of subspecialty, radiographic, and operative services. The ED serves approximately 65,000 patients per year. Every cardiac arrest patient is managed by a resuscitation team consisting of emergency physicians and residents. The advanced cardiac life support (ACLS) protocol is based on current AHA guidelines. All imaging studies were ordered based on clinician gestalt and practice and not dictated by protocol.

### Study design, population, and outcome measures

We performed a retrospective observational descriptive study of all adult patients (age ≥ 18 years) who presented to the UF Health ED during a 6-year period beginning on January 1, 2013 and ending on December 15, 2018. We included patients with a chief complaint or ED diagnosis of cardiac arrest, ventricular fibrillation, ventricular tachycardia, asystole, or pulseless electrical activity.

A list of patients meeting inclusion criteria was queried from the patient electronic medical record (Epic, Epic Systems, Madison, WI). Data points that were collected included age, gender, chief complaint, initial presenting rhythm, location of arrest (prehospital vs. ED), whether return of spontaneous circulation (ROSC) was achieved, where ROSC was achieved (prehospital vs. ED), final ED disposition (admit, death, discharge), ED radiology results, and subsequent interventions (e.g. blood product administration, surgical consultations, subspecialty consultations, other procedural interventions).

We excluded patients that (1) were transferred from another hospital, (2) had a current valid do-not-resuscitate (DNR) order in place prior to ROSC, (3) suffered cardiac arrest as a result of trauma, (4) had a chart created in error, or (5) were incorrectly labelled as cardiac arrest (e.g. non-sustained VT, VT with a pulse, etc.). Patients who did not achieve sustained ROSC were excluded.

Only cross-sectional imaging studies performed in the ED were included for analysis. Each radiographic finding in the final impression of each radiology report was tallied. Using a predefined scale to characterize the acuity of radiographic findings (Box 1), each clinical radiology report was scored by two independent and blinded reviewers. After initial scoring, each score was subsequently reviewed and verified by the lead author for accuracy. In situations where an imaging report had multiple findings of varying acuity levels, the highest acuity finding would take precedence (e.g. A > B > C or D > E). The reviewers consisted of DZC and JDW who were second-year medical students, AA who was a first-year medical student, and CWH (lead author) who has been an emergency physician for seven years and is board-certified in emergency medicine.

Box 1 **Predefined scale to characterize the acuity of findings based on radiographic results.**

**Table:** 

Acuity of Radiographic Findings	Radiology Result
A	Radiographic evidence of a condition that likely contributed to cardiac arrest
B	Radiographic evidence of sequelae secondary to cardiac arrest or resuscitative efforts
• B1	Fractures (e.g. sternal, rib, etc.) as a result of resuscitative efforts
• B2	Any other sequelae (besides fractures) secondary to cardiac arrest or resuscitative efforts (e.g. hypoxic brain injury, cerebral oedema, pulmonary contusion, shock liver, etc.)
	
C	Radiographic findings suggestive of chronic conditions
D	Radiographic findings of uncertain significance or incidental findings
E	No acute radiographic findings or abnormalities

Alt-text: Box 1

Clinician and clinical re-evaluation notes were reviewed; specialty consultations and interventions which were directly influenced by cross-sectional imaging findings were tallied. For example, a tube thoracostomy that was performed based on plain film radiographs and subsequently shows up on CT imaging was not counted, but a tube thoracostomy that was performed based on CT imaging was counted.

The outcome measures for this study included a descriptive analysis of post-cardiac arrest patients, including demographics, radiographic results, and acuity of radiographic findings.

Institutional Review Board (IRB) approval (#201802862) was obtained prior to the initiation of the study.

### Statistical analysis

We entered data from the study into a REDCap (Research Electronic Data Capture, University of Florida, Gainesville, FL) database. Descriptive statistics (means, frequencies, and percentages) were used to characterize the study population. All clinical data were extracted from REDCap to Stata (StataCorp. 2017. Stata Statistical Software: Release 15. College Station, TX: StataCorp LLC).

## Results

**Fig. 1:**
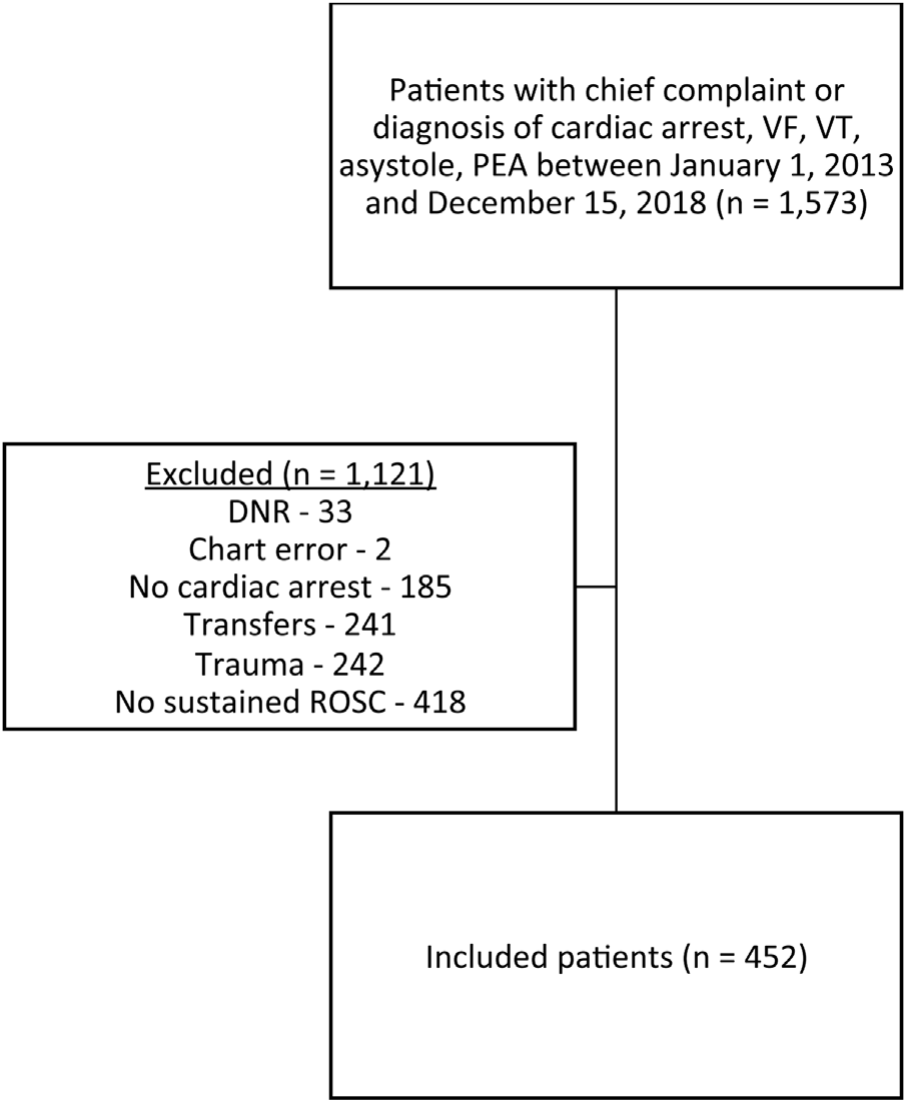
Flowchart of inclusion and exclusion criteria. DNR, do not resuscitate; PEA, pulseless electrical activity; ROSC, return of spontaneous circulation; VF, ventricular fibrillation; VT, ventricular tachycardia.

We identified a total of 1573 patients with a chief complaint or diagnosis of cardiac arrest, VF, VT, asystole, or PEA that presented to the UF Health ED between January 1, 2013 and December 15, 2018. We excluded 1121 patients: 33 patients had a current valid DNR order in place; 2 charts were created in error; 185 patients did not experience cardiac arrest; 241 patients were transferred from other hospitals; 242 patients were associated with trauma; and 418 did not achieve sustained ROSC. Thus, 452 patients were included for analysis (Fig. 1). Complete patient characteristics are reported in Table 1.

**Table 1:** Descriptive statistics for all patients that experienced cardiac arrest and achieved sustained return of spontaneous circulation. ED, emergency department; EMS, emergency medical services; LOS, length of stay; PEA, pulseless electrical activity; VF, ventricular fibrillation; VT, ventricular tachycardia.

	Total (*N* = 452)
Age, mean (SD)	62.0 (15.6)
Age groups	
≤ 60 years, *n* (%)	206 (45.6)
> 60 years, *n* (%)	246 (54.4)
Gender	
Female, *n* (%)	168 (37.2)
Male, *n* (%)	284 (62.8)
Initial Rhythm	
Asystole, *n* (%)	99 (21.9)
PEA, *n* (%)	171 (37.8)
VF, *n* (%)	97 (21.5)
VT, *n* (%)	25 (5.5)
Unknown, *n* (%)	60 (13.3)
ED/EMS Arrest	
ED, *n* (%)	69 (15.3)
EMS, *n* (%)	383 (84.7)
ED Disposition	
Admit, *n* (%)	414 (91.6)
Death, *n* (%)	33 (7.3)
Discharge, *n* (%)	3 (0.7)
Transfer, *n* (%)	2 (0.4)
ED Disposition time, mean (SD), *h*	5.9 (7.8)
LOS, mean (SD), *d*	7.7 (20.0)

Of 452 patients, 316 received cross-sectional imaging and 136 did not receive cross-sectional imaging. 179 patients received more than one cross-sectional imaging study. Common imaging findings of the head, chest, and abdomen are shown in Table 2. Specialty consultations and interventions which were directly influenced by cross-sectional imaging findings are also shown in Table 2. A complete table of all imaging findings is included in Supplemental Table 1.

**Table 2:** Common cross-sectional radiographic findings of the head, chest, abdomen, and pelvis, and subsequent consultations and interventions. For a complete table, refer to *Supplemental* Table 1. ENT, otolaryngology; ICP, intracranial pressure; IR, interventional radiology; IVH, intraventricular haemorrhage; SAH, subarachnoid haemorrhage; SDH, subdural hematoma.

Table 2–1. Common cross-sectional radiographic findings of the head			
Finding	No.	%	Interventions (e.g. consultations, procedures) (*n*)
No acute intracranial abnormalities	185	62.1	
Hypoxic/ischaemic injury, loss of grey-white differentiation	58	19.5	Neurosurgery consult (3)
Diffuse cerebral oedema	47	15.8	Neurosurgery consult (2)
Advanced chronological age, cerebral volume loss	39	13.1	
Atherosclerotic disease, chronic ischemic changes, remote infarct	37	12.4	
Herniation	23	7.7	Neurosurgery consult (10)
Haemorrhage, IVH, SDH, SAH	14	4.7	Neurosurgery consult (12)
Soft tissue swelling	13	4.4	
Evolving infarction	9	3.0	Neurosurgery consult (2)
Nonspecific hypodense focus	9	3.0	
**Total number of CT head**	**298**		

Table 2-2. Common cross-sectional radiographic findings of the chest			
Finding	No.	%	Interventions (e.g. consultations, procedures) (*n*)
Bibasilar airspace opacities	138	75.0	
Rib fracture, sternal fracture	93	50.5	
Aspiration	87	47.3	
Pleural effusion	43	23.4	Tube thoracostomy (2)
Pulmonary oedema	32	17.4	
Pneumothorax, pneumomediastinum	24	13.0	Tube thoracostomy (8)
Endotracheal tube, catheter issue	18	9.8	
Congestive heart failure, anasarca, ascites	17	9.2	
Cardiomegaly	15	8.2	
Coronary artery disease	14	7.6	
Pulmonary embolism and right heart strain	14	7.6	Tissue plasminogen activator (5), IR consult (1), Haematology consult (1)
**Total number of CT chest**	**184**		

Table 2-3. Common cross-sectional radiographic findings of the abdomen and pelvis			
Finding	No.	%	Interventions (e.g. consultations, procedures) (*n*)
No acute abdominal abnormalities	25	22.1	
Cirrhosis, ascites, anasarca	21	18.6	Vitamin K and Prothrombin complex concentrate (1)
Shock bowel	15	13.3	Surgery consult (1)
Renal infarct, disease, cysts	14	12.4	
Cholelithiasis	14	12.4	
Miscellaneous/chronic conditions (cyst, cancer, adenoma)	14	12.4	
Nonspecific bowel abnormalities	13	11.5	
Periportal oedema	8	7.1	
Findings of recent line placement, Foley placement, complications (e.g. contrast extravasation/pseudoaneurysm)	7	6.2	
Nonspecific hepatic irregularity, steatosis	7	6.2	
Haemoperitoneum, viscus injury	6	5.3	Surgery consult (2), IR consult (1)
**Total number of CT abdomen/pelvis**	**113**		

For the 316 patients that received cross-sectional imaging, the acuity of each radiographic study based on our predefined scale is listed in Table 3. Cross-sectional imaging identified acute findings that are likely related to cardiac arrest in the head (4.4%), chest (12.5%), and abdomen (5.3%). Altogether, 42 (7.1%) radiographic studies had findings that likely contributed to SCA. A breakdown of radiology studies per patient and findings that likely contribute to cardiac arrest per scan is shown in Table 4. Eighty (13.4%) studies (head [n = 38, 12.8%], chest [n = 26, 14.1%], abdomen [n = 16, 14.2%]) resulted in a change of clinical care (e.g. specialty consultation, medication administration, or procedures).

**Table 3:** Acuity of radiographic findings for all patients that experienced cardiac arrest, achieved return of spontaneous circulation, and received cross-sectional imaging in the emergency department. CT, computed tomography.

Radiographic findings	CT Head – *N* (%)	CT Chest – *N* (%)	CT Abdomen – *N* (%)	Total – *N* (%)
				
Findings that likely contributed to cardiac arrest	13 (4.4%)	23 (12.5%)	6 (5.3%)	42 (7.1%)
				
Fractures resulting from resuscitative efforts (e.g. rib, sternal)	0 (0)	82 (44.6%)	1 (0.9%)	83 (13.9%)
				
Other sequelae (besides fractures) resulting from resuscitative efforts	73 (24.5%)	7 (3.8%)	23 (20.4%)	103 (17.3%)
				
Chronic condition	81 (27.2%)	8 (4.3%)	27 (23.9%)	116 (19.5%)
				
Uncertain significance or incidental findings	19 (6.4%)	62 (33.7%)	33 (29.2%)	114 (19.2%)
				
No acute abnormality	112 (37.6%)	2 (1.1%)	23 (20.4%)	137 (23.0%)
				
Total	298	184	113	595

**Table 4:** Radiology studies performed per patient and the number of studies with findings that likely contributed to cardiac arrest. CT, computed tomography.

Radiology studies performed per patient	*n* (%)	Number of studies with findings likely contributing to cardiac arrest
		
CT head	126 (39.9%)	8
		
CT chest	9 (2.8%)	1
		
CT abdomen	2 (0.6%)	0
		
CT head and chest	68 (21.5%)	head 2 chest 7
		
CT head and abdomen	4 (1.3%)	0
		
CT chest and abdomen	7 (2.2%)	0
		
CT chest, abdomen, pelvis	100 (31.6%)	head 2 chest 14 abdomen 5 head, chest, abdomen 1
		
Total	316	80

## Discussion

The purpose of this study was to perform a descriptive analysis of cross-sectional radiographic findings after cardiac arrest. Differences between the number of acute findings likely related to cardiac arrest and findings unlikely related to cardiac arrest in Table 2, Table 3 are due to the grading process; imaging reports with multiple findings of varying acuity levels were graded based on the highest acuity finding (e.g. A > B > C or D > E).

Our data demonstrate that acute conditions are frequently discovered on cross-sectional imaging studies. These findings may include conditions that are the proximate cause of cardiac arrest (e.g. intracranial haemorrhage, pulmonary embolism, aortic dissection), acute sequelae of cardiac arrest and resuscitation (e.g. hypoxic-ischaemic cerebral injury, diffuse cerebral oedema, rib and sternal fracture, shock bowel, renal infarction), or both (e.g. aspiration, evolving intracranial infarction, cerebral herniation, pneumothorax, pulmonary oedema). Quite frequently, it is impossible to determine clinically whether a condition existed prior to cardiac arrest or was the result of resuscitative efforts. In the peri-arrest resuscitative phase, however, the temporal relationship is not as important as the awareness that such a condition exists and will require immediate medical or surgical management.

Moreover, imaging can reveal complications that arise from resuscitative efforts, such as iatrogenic rib fracture and sternal fracture (n = 93, 50.5%), endotracheal tube and catheter misplacement (n = 18, 9.8%), mediastinal hematoma (n = 5, 2.7%), and iatrogenic injuries from line placement (e.g. retroperitoneal hematoma, vascular pseudoaneurysm, active extravasation) (n = 7, 6.2%), or findings of blunt trauma from CPR (e.g. pneumothorax, pneumoperitoneum) requiring urgent intervention. Finally, cross-sectional imaging may reveal findings that are helpful in prognostication and goals-of-care discussions with family.

Notably, only one MRI was performed after an abnormal CT of the head to further evaluate a patient with intracranial ischaemia. The solitary MRI reflects the limited use and perceived lack of utility of MRI after cardiac arrest.

Kürkciyan et al. concluded that the cause of cardiac arrest is not as easily predictable as anticipated, and clinical suspicion and physician gestalt remain a large and influential factor in the post-arrest resuscitation phase.^21^ Early knowledge of the origin of cardiac arrest can directly influence cause-specific treatment, indirectly influencing patient outcomes.^21^ Imaging is useful in identifying pathology that caused cardiac arrest or pathology that resulted from cardiac arrest resuscitation, thereby influencing downstream management decisions.

Previous research has evaluated the diagnostic utility of non-invasive imaging modalities.[1], [23], [29], [30] Unfortunately, many of these studies are limited by sample size and lack robust diagnostic accuracy. In Petek et al.’s systematic review of 17 articles evaluating the diagnostic yield of non-invasive imaging in patients after OHCA, potential causes of OHCA were found in 8-54% of patients following head, chest, and/or abdominal CT. Changes to clinical management following imaging varied from 25 to 60%.^23^ The authors conclude that many questions remained regarding the clinical application of CT in identifying non-cardiac causes in OHCA survivors.

In a retrospective study evaluating the diagnostic yield of whole-body CT (WBCT) in OHCA patients, acute pathology was found in the head (15%), chest (70%), and abdomen (6%), although whether these findings were the proximate cause of cardiac arrest is unclear.^6^ Chelly et al. found that the cause of cardiac arrest was identified by CT imaging in 20% of cases, although the identification of the cause by CT was an independent factor associated with ICU mortality.^1^ Although the prognosis of OHCA patients is poor, the identification and treatment of the underlying cause remains a cornerstone of post-cardiac arrest care;[1], [31] this will be increasingly important as cardiac arrest and post-arrest care continue to advance and outcomes improve.

One of the major limitations of this study is its retrospective observational nature, which precluded a complete database with Utstein variables. Given the immediate and unpredictable nature of cardiac arrest and the numerous causes, a prospective evaluation would be difficult. Other limitations include referral bias and survival bias due to the specific patient population being studied.

The role of imaging in cardiac arrest continues to be relegated to the clinician based on clinician gestalt with no formal guidelines or suggestions according to particular patient populations and presentations. Future studies will be needed to elucidate specific situations requiring cross-sectional imaging. Future studies may also look at long-term outcome data, ED length-of-stay, hospital length-of-stay, and goals-of-care discussions based on imaging acuity.

## Conclusion

Given the clinical uncertainty, relative patient instability, and variability in patient conditions during the post-cardiac arrest resuscitation phase, cross-sectional imaging frequently reveals diagnostic findings that may impact downstream medical decisions.

## Authorship contributions

CWH and TKB conceived the manuscript concept. CWH, DZC, JDW, and AA participated in data collection. MABC assisted with data analysis. CWH wrote the first draft. All authors proofread and approved the final version of the manuscript.

## Funding

This research did not receive any specific grant from funding agencies in the public, commercial, or not-for-profit sectors.

## Declaration of interests

The authors declare that they have no known competing financial interests or personal relationships that could have appeared to influence the work reported in this paper.

## Conflicts of interest

None.

## Disclosures

The authors have indicated they have no financial relationships relevant to this article to disclose.
