# Supplementary material for: A descriptive analysis of cross-sectional imaging findings in patients after non-traumatic sudden cardiac arrest
Source: Resusc Plus. 2021 Jan 28;5:100077. doi: 10.1016/j.resplu.2021.100077 (PMC8244399; doi:10.1016/j.resplu.2021.100077)
Supplement: Supplementary file 1 [file mmc1.docx]

| Supplemental Table 1-1. Cross-sectional radiographic findings of the head | | | |
| --- | --- | --- | --- |
| Finding | No. | % | Interventions (e.g. consultations, procedures) (*n*) |
| No acute intracranial abnormalities | 185 | 62.1 |  |
| Hypoxic/ischaemic injury, loss of grey-white differentiation | 58 | 19.5 | Neurosurgery consult (3) |
| Diffuse cerebral oedema | 47 | 15.8 | Neurosurgery consult (2) |
| Advanced chronological age, cerebral volume loss | 39 | 13.1 |  |
| Atherosclerotic disease, chronic ischemic changes, remote infarct | 37 | 12.4 |  |
| Herniation | 23 | 7.7 | Neurosurgery consult (10) |
| Haemorrhage, IVH, SDH, SAH | 14 | 4.7 | Neurosurgery consult (12) |
| Soft tissue swelling | 13 | 4.4 |  |
| Evolving infarction | 9 | 3.0 | Neurosurgery consult (2) |
| Nonspecific hypodense focus | 9 | 3.0 |  |
| Masses (aneurysm, meningioma) | 8 | 2.7 | Neurosurgery consult (2), ENT consult (1) |
| Head and neck cancer | 7 | 2.3 | Neurosurgery consult (1) |
| Facial fracture (acute/chronic) | 7 | 2.3 |  |
| Hydrocephalus | 6 | 2.0 | Neurosurgery consult (3) |
| Postsurgical changes | 3 | 1.0 |  |
| Cervical fracture | 2 | 0.7 | Neurosurgery consult (1) |
| Facial abscess | 2 | 0.7 |  |
| Subdural hygroma | 1 | 0.3 |  |
| Findings of elevated ICP | 1 | 0.3 |  |
| Atlantooccipital dislocation | 1 | 0.3 | Neurosurgery consult (1) |
| **Total number of CT head** | **298** |  |  |

| Supplemental Table 1-2. Cross-sectional radiographic findings of the chest | | | |
| --- | --- | --- | --- |
| Finding | No. | % | Interventions (e.g. consultations, procedures) (*n*) |
| Bibasilar airspace opacities | 138 | 75.0 |  |
| Rib fracture, sternal fracture | 93 | 50.5 |  |
| Aspiration | 87 | 47.3 |  |
| Pleural effusion | 43 | 23.4 | Tube thoracostomy (2) |
| Pulmonary oedema | 32 | 17.4 |  |
| Pneumothorax, pneumomediastinum | 24 | 13.0 | Tube thoracostomy (8) |
| Endotracheal tube, catheter issue | 18 | 9.8 |  |
| Congestive heart failure, anasarca, ascites | 17 | 9.2 |  |
| Cardiomegaly | 15 | 8.2 |  |
| Coronary artery disease | 14 | 7.6 |  |
| Pulmonary embolism and right heart strain | 14 | 7.6 | Tissue plasminogen activator (5), IR consult (1), Haematology consult (1) |
| Cavitation, mass | 13 | 7.1 | Surgery consult (1) |
| Pulmonary artery hypertension | 13 | 7.1 |  |
| Thyroid nodules | 8 | 4.3 | Surgery consult (1) |
| Emphysema | 8 | 4.3 |  |
| Mediastinal hematoma | 5 | 2.7 |  |
| Chest wall contusion | 4 | 2.2 |  |
| Thoracic aortic aneurysm | 3 | 1.6 |  |
| Pericardial effusion | 2 | 1.1 |  |
| Pulmonary artery haemorrhage | 1 | 0.5 | Surgery consult (1) |
| Superior vena cava syndrome | 1 | 0.5 |  |
| Upper gastrointestinal bleeding | 1 | 0.5 | IR consult (1), Gastroenterology consult (1), Surgery consult (1) |
| Internal mammary artery haemorrhage, haemomediastinum | 1 | 0.5 | IR consult (1), Cardiothoracic surgery consult (1) |
| Pulmonary fibrosis | 1 | 0.5 |  |
| Spinal compression fracture | 1 | 0.5 |  |
| Remote myocardial infarction, ventricular aneurysm | 1 | 0.5 |  |
| Adrenal nodule | 1 | 0.5 |  |
| Aortic dissection | 1 | 0.5 | Surgery consult (1) |
| Right heart strain | 1 | 0.5 |  |
| Renal aneurysm | 1 | 0.5 |  |
| Pericardial cyst | 1 | 0.5 |  |
| Pneumopericardium | 1 | 0.5 |  |
| **Total number of CT chest** | **184** |  |  |

| Supplemental Table 1-3. Cross-sectional radiographic findings of the abdomen and pelvis | | | |
| --- | --- | --- | --- |
| Finding | No. | % | Interventions (e.g. consultations, procedures) (*n*) |
| No acute abdominal abnormalities | 25 | 22.1 |  |
| Cirrhosis, ascites, anasarca | 21 | 18.6 | Vitamin K and Prothrombin complex concentrate (1) |
| Shock bowel | 15 | 13.3 | Surgery consult (1) |
| Renal infarct, disease, cysts | 14 | 12.4 |  |
| Cholelithiasis | 14 | 12.4 |  |
| Miscellaneous/chronic conditions (cyst, cancer, adenoma) | 14 | 12.4 |  |
| Nonspecific bowel abnormalities | 13 | 11.5 |  |
| Periportal oedema | 8 | 7.1 |  |
| Findings of recent line placement, Foley placement, complications (e.g. contrast extravasation/pseudoaneurysm) | 7 | 6.2 |  |
| Nonspecific hepatic irregularity, steatosis | 7 | 6.2 |  |
| Haemoperitoneum, viscus injury | 6 | 5.3 | Surgery consult (2), IR consult (1) |
| Ileus | 6 | 5.3 |  |
| Hernia | 5 | 4.4 | Surgery consult (1) |
| Pneumoperitoneum | 4 | 3.5 | Surgery consult (1) |
| Atherosclerotic disease, decreased graft patency | 4 | 3.5 | Vascular surgery consult (1) |
| Postsurgical (chronic) change | 3 | 2.7 |  |
| Nonspecific free fluid in abdomen | 3 | 2.7 | Surgery consult (1) |
| Abdominal wall/rectus sheath haemorrhage | 3 | 2.7 |  |
| Vertebral fracture | 3 | 2.7 |  |
| Portal venous gas | 2 | 1.8 | Surgery consult (1) |
| Duodenitis | 2 | 1.8 |  |
| Retroperitoneal hematoma | 2 | 1.8 | Surgery consult (1) |
| Splenic subcapsular hematoma | 2 | 1.8 | Surgery consult (1) |
| Intussusception | 1 | 0.9 |  |
| Impaction | 1 | 0.9 |  |
| Soft tissue changes | 1 | 0.9 |  |
| Deep venous thrombosis | 1 | 0.9 |  |
| Pancreatitis | 1 | 0.9 |  |
| Diverticulitis | 1 | 0.9 |  |
| Aneurysm (unruptured) | 1 | 0.9 | IR consult (1) |
| Pneumobilia | 1 | 0.9 |  |
| Lymphadenopathy | 1 | 0.9 |  |
| Splenic infarction | 1 | 0.9 |  |
| Small bowel obstruction | 1 | 0.9 |  |
| Splenomegaly | 1 | 0.9 |  |
| Pneumatosis | 1 | 0.9 |  |
| Genitourinary (cystitis, hydroureter, hydronephrosis) abnormalities | 1 | 0.9 |  |
| Pleural effusion | 1 | 0.9 |  |
| Pneumonia | 1 | 0.9 |  |
| Hepatocellular carcinoma rupture | 1 | 0.9 | Surgery consult (1), IR consult (1) |
| Portal venous thrombosis | 1 | 0.9 |  |
| Haemorrhage from intraabdominal artery | 1 | 0.9 | Surgery consult (1) |
| **Total number of CT abdomen/pelvis** | **113** |  |  |

Supplemental Table 1. Cross-sectional radiographic findings of the head, chest, abdomen, and pelvis, and subsequent consultations and interventions. ENT, otolaryngology; ICP, intracranial pressure; IR, interventional radiology; IVH, intraventricular haemorrhage; SAH, subarachnoid haemorrhage; SDH, subdural hematoma.
